# The Concept of Pain in Children Attending Primary School: Implications for School-Based Pain Education

**DOI:** 10.1155/prm/3198988

**Published:** 2025-01-16

**Authors:** Talita Odendaal, Ina Diener, Quinette Abegail Louw, Dawn Verna Ernstzen

**Affiliations:** Division of Physiotherapy, Department of Health and Rehabilitation Sciences, Stellenbosch University, P.O. Box 241, Cape Town 8000, South Africa

## Abstract

**Introduction:** A child's concept of pain comprises their understanding of what pain is, the purpose of pain, and biological processes underpinning pain. The concept of pain can influence pain experiences, pain beliefs, and pain-related behaviour. This study aimed to assess the concept of pain among children attending primary schools in Gqeberha in the Eastern Cape of South Africa. A secondary aim was to explore demographic and pain-related information that may contribute to a child's concept of pain.

**Materials and Methods:** A cross-sectional study with an analytic component was used. The cross-culturally adapted Concept of Pain Inventory (COPI) was used to assess the concept of pain amongst 12-year-old school-attending children. Children from seven primary schools that were selected via stratified random sampling participated. Participants completed the adapted COPI and a sociodemographic questionnaire. Descriptive statistics and inferential analysis were used to analyse the data.

**Results:** There were 119 participants. Participants' concept of pain partially aligned with contemporary pain science (mean = 34.39 out of 56; standard deviation = 6.49), indicating a partial understanding of the factors influencing pain. Demographic factors and pain-related information investigated did not influence participants' concept of pain. However, differences in the concept of pain were observed amongst participants from different schools.

**Conclusions:** Participants had a developing concept of pain that was partially aligned with contemporary pain science. Participants had pain knowledge strengths and gaps that can be used to develop a tailored school-based pain education intervention for them. There were indications that contextual factors may have influenced the participants' concept of pain. Further studies to explore socioenvironmental factors that influence pain knowledge in children are recommended.

## 1. Introduction

Pain is a common experience in childhood, yet it may affect a child's functioning and participation in life roles. Acute pain in childhood may be from bumps, bruises or immunizations, and children may also experience persistent pain such as headache, musculoskeletal pain or abdominal pain. The global prevalence of chronic pain amongst children and adolescents is 20.8% (95% CI: 19.2–22.4) [1]. Similarly, a recent review estimated the pooled mean prevalence of paediatric general chronic pain in low- and middle-income countries (LMIC) to be 20% [2]. Pain can interfere with a child's daily activities and can affect school attendance, academic performance and participation in recreational activities leading to social exclusion. Additionally, chronic pain can lead to pain-related anxiety and depression, ultimately affecting a child's quality of life [2, 3]. The impact of pain on the functioning of a child necessitates healthcare prioritisation.

Pain is a multidimensional experience; therefore, optimal management of acute and chronic pain in children requires a multimodal approach, in which educational interventions can play a key role [4, 5]. Pain education can be a preventative strategy to limit the transition of acute pain to chronic pain by enhancing a child's concept of pain, their understanding and beliefs about pain and developing their ability to cope with pain [6]. A person's concept of pain comprises an understanding of what pain is, the purpose of pain, and the biological processes underlying pain [7]. Education about the above aspects is important. Contemporary pain science purports that pain is a biopsychosocial phenomenon that is influenced by multiple factors, comprising personal factors (e.g., age and gender), biological factors (e.g., nociception and genetics), psychological factors (e.g., cognitions, beliefs and emotions), behavioural factors (e.g., coping responses) and sociocontextual factors (e.g., family, culture and environmental factors) [6, 8, 9]. These biopsychosocial factors play a role in the pain experience, pain processing and the concept of pain as well pain management [6, 9, 10]. An understanding of the factors influencing pain can contribute to the development of accurate beliefs about pain and can optimise pain coping mechanisms. Such understanding can furthermore decrease pain-related anxiety, positively influencing physical movement and functional outcomes, and promote self-management of pain [5, 6]. Therefore, pain education is a key to enhance pain knowledge, to decrease the threat value of pain and to improve the management of pain in children. Pain education can be provided to children with pain conditions as a management strategy and can also be provided to children as a preventative strategy, for example, in school settings in the format of a public health approach.

Public health approaches focussing on pain education are an important strategy to enhance pain literacy in children [11]. School-based pain education initiatives in the United Kingdom (UK), United States of America (USA), and Spain showed promising results in positively influencing children's beliefs and knowledge about pain and approaches to physical activity when in pain [11–15]. These school-based pain education initiatives comprised pain neuroscience education (PNE) [11–15], which covered understanding pain as a biopsychosocial phenomenon, while another [15] focussed on understanding what pain is, the factors that influence pain, and how pain is managed. Pain education interventions are therefore important to facilitate pain-related knowledge which can play a role to limit the long-term consequences of pain.

For pain education to be effective, healthcare professionals need to understand a child's conceptualisation of pain and be aware of the child's knowledge needs and possible misconceptions about pain [16]. Outcome measures, such as the Concept of Pain Inventory (COPI), have been developed to assess children's conceptualisation of pain [17]. The COPI aims to assess a child's knowledge about the purpose of pain, the biological processes underpinning pain, and how pain works [17]. Congruently, other studies have used different tools to ascertain children's concept or knowledge about pain, for example, the Conceptualization of Pain Questionnaire (COPAQ) [18] and the Neurophysiology of Pain Questionnaire (NPQ) [12]. The results of these outcome measurement tools can be used to tailor educational interventions for children and to monitor changes in knowledge about pain.

Despite the significance of a child's concept of pain, little research has been conducted on the topic, in the African context. Since socioenvironmental factors may impact children's knowledge and experience of pain [6, 19, 20], research regarding children's concept of pain in a multicultural society such as South Africa (SA) can inform contextually relevant interventions, facilitating optimised pain management and the well-being of children. The objective of the study was to ascertain children's concept of pain in a SA setting and to investigate demographic and pain-related information that may contribute to children's concept of pain.

## 2. Materials and Methods

### 2.1. Study Design

A descriptive cross-sectional study [21] with an analytical component was conducted to determine primary school-attending children's concept of pain, using the COPI. The COPI results are described, and in the analytical component, the correlation between COPI scores and the demographic and pain-related information is explored. The manuscript was prepared using the STrengthening the Reporting of Observational studies in Epidemiology (STROBE) checklist for observational studies as guideline (Supporting file 1).

### 2.2. Study Setting

The study was conducted in Gqeberha, in the Eastern Cape Province of SA. Gqeberha has a population of 1,152,115 inhabitants with isiXhosa (78,8%), Afrikaans (10,5%), and English (5.6%) as the main languages spoken in the area [22].

### 2.3. Sampling Strategy

The study population comprised 12-year-old children attending primary schools in the above setting. In the SA school system, Grade 6 is the expected grade 12-year-old children. This age group was selected because it represents a vital developmental time in which children can grasp complicated ideas and participate in meaningful dialogues [7, 11].

The schools eligible to participate were selected using stratified random sampling. Children at the abovementioned selected schools could participate if they met the inclusion criteria and were willing to participate [23]. About 7.3% (8183.67) of school attending children were in Grade 6, and the estimated class size was 30.65 children per class per school in Gqeberha [24]. The city, Gqeberha had 232 public and 35 private primary schools at the time of the study [25]. A 50% stratified random sample of schools was selected via MS Excel [26]. A total of 14 primary schools in Southern and Western Gqeberha were recruited to participate (eight public urban schools, two public farm schools (semiurban) and four private schools). Therefore, seven schools were selected, of which four were public urban, two were private urban and one a farm school. One school declined to participate, and the next school on the random generated list was invited to participate. The different categories and locations of the schools were chosen to include different socioenvironmental contexts, which may influence knowledge and beliefs about pain [10, 27]. The language of tuition at the selected schools was English, Afrikaans, or isiXhosa [28].

Children eligible to participate had to be 12 years old at the time of data collection, attend a participating school in Gqeberha, be willing to participate and be proficient in English, Afrikaans or isiXhosa. Participating children needed to provide assent, and their parents/caregivers had to provide informed consent. Children who had previously received care from the primary investigator (PI) (who is a physiotherapist) were excluded from participating [23].

### 2.4. Sample Size Calculation

A biostatistician assisted with the sample size calculation. The primary objectives were used to estimate the sample size. The secondary objective was exploratory and was not used in the sample size calculation. Therefore, a pragmatic estimation based on the number of classes in the eligible schools was used as follows: four classes in four public urban schools (4 × 4 × 30.65), three classes in two private schools (3 × 2 × 30.65), and one class in one farm schools (1 × 30.65). An estimation of 122.6 Grade 6 children in public urbans, 91.95 in private schools, and 30.65 in the farm school was used to calculate a finite population of 705 12-year-old children. The estimated precision for a one-mean 95% confidence interval from a scale score with a standard deviation (SD) of 1 is 0.45 and was calculated using a minimum sample size of 82.

### 2.5. Data Collection Tools

Two data collection instruments were used, namely, a sociodemographic questionnaire (SDQ) and the COPI. The SDQ collected demographic information on age, gender, home language, and pain-related information that could potentially influence a child's concept of pain. The health-related information (Table 1) comprised current pain, previous pain, healthcare accessed for pain, and family members with pain [29, 30].

The concept of pain was assessed using the COPI [7], a 14-item tool which was developed for children aged 8 to 12 years. Each item is scored on a five-point Likert scale (0 = “*strongly disagree*”; 1 = *disagree*; 2 = *unsure*; 3 = *agree*; and 4 = “*strongly agree*). The COPI is scored out of 56, with higher scores reflecting greater alignment of the child's concept of pain with contemporary pain science [7]. The COPI displayed adequate psychometric properties with acceptable internal consistency (*α* = 0.775) and moderate test-retest reliability (intraclass correlation coefficient = 0.55; 95% CI, 0.37–0.68) in children [7]. The Turkish version of the COPI was found to have acceptable internal consistency (*α* = 0.78) and an intraclass correlation coefficient of 0.93 [31].

The COPI was cross-culturally adapted for the SA context, since the COPI was originally developed for English-speaking children in Australia [7]. A cross-cultural adaptation framework [32] was used to assess and adapt the conceptual item and semantic equivalence of the original COPI to the SA context. The cross-cultural adaptation was completed in four steps. In step one, a multidisciplinary panel of SA paediatric pain experts assessed the relevance and acceptability of the COPI items for the SA context. The panel also evaluated whether any of the items may trigger emotional responses in children that may require them to receive counselling. In step two, the adapted COPI, was translated into Afrikaans and isiXhosa by professional translators at the Stellenbosch University (SU) Language Centre. In step three, the adapted and translated COPI were evaluated by the same experts as in step one, some of whom were proficient in Afrikaans and isiXhosa to ascertain its equivalence to the adapted English COPI. In step four, a pilot study with a group of three 12-year-old children was conducted, to ascertain the usability of the adapted COPI in the Afrikaans, English, and isiXhosa. The COPI was adapted by clarifying the meaning of some words and several semantic adaptations were made. There was consensus regarding the item equivalence (relevance and acceptability) and semantic equivalence between the original and adapted COPI. No conceptual (cultural) adaptations were advised by the panel [33]. However, further exploration on the conceptual (cultural), operational (mode), and measurement (psychometric) equivalences is advised. The SDQ, assent and consent forms were also translated to Afrikaans and isiXhosa by the SU Language Centre.

### 2.6. Procedures

The study proposal was approved by the Department of Education of the Eastern Cape, SA and the SU Health Research Ethics Committee (SU HREC) (S20/10/276), which included ministerial consent for nontherapeutic health research with minors. Data were collected from September to October 2021, when in-person access to schools for research purposes was restricted due to COVID-19 regulations. These restrictions impacted data collection procedures as the researcher was not allowed on the school premises and had to rely on electronic communication, as well as research assistance from the school principal and teachers, as elaborated below.

A study information leaflet and an invitation to participate in the study were emailed to the principals of the schools that were sampled. Upon principals accepting the invitation, a teacher that was willing to assist with the study procedures was recruited and appointed by the principal. A convenient day and time were scheduled to deliver age-appropriate study information leaflets and assent and consent forms, in the language medium of the school (Afrikaans, English and/or isiXhosa). The researcher wore a mask and gloves when handling documents due to COVID-19 precautions (National Archives and Record Services of SA, 2020). Printed documents were quarantined for 72 h in a sealed box before the appointed teacher distributed the documents to selected children. The teacher wore gloves, supplied by the researcher, when handling the documents. Each school had their COVID-19 guidelines (Department of Basic Education, 2020), which were adhered to as per school practice.

The PI explained the research procedure telephonically to the teacher. The teacher explained the purpose of the study, and the meaning of voluntary participation and anonymity, as well as the eligibility criteria to the children (e.g., the age restriction and the role of the researcher). The teacher also informed the children, that if they had received physiotherapy treatment form the researcher, they would not be eligible to participate. The researcher was available on the telephone for any additional clarifications needed. Thereafter, the information leaflet, assent and informed consent documents (in their language of choice) were handed to the children meeting the inclusion criteria and interested to participate, to discuss with their parents/caregivers. The provided documentation assured carergivers that the participants' details would be kept confidential. The contact details of the PI as well as those of SU-HREC were provided on the informed consent form if a parent/guardian had any questions related to the study.

One week after the invitation, the teacher distributed the two self-report questionnaires to the children who had provided signed assent and consent forms. The forms were completed in the classroom, while the PI was available telephonically, to answer any questions. To ensure confidentiality, no personal identification information was requested from the participants and each questionnaire had a unique reference number. Each child was asked to put their signed consent and assent forms and later their completed questionnaires into a box supplied by the researcher. The documents were left in quarantine for 72 h before data processing. The PI collected the sealed postbox with the completed questionnaires from the school. The PI (via the teacher) thanked the participants for their participation and provided them with a stationery gift.

### 2.7. Data Management and Analysis

The PI electronically captured and stored the data, on an MS Excel datasheet. IMB SSPS Version 28 was used for data analysis. The mean and SD were calculated for numerical outcomes, and frequency and percentage with categorical outcomes. The total score from the adapted COPI was used as the dependent variable regarding the concept of pain. All questionnaires were complete.

For the secondary objective, parametric and statistical tests were used to compare scores between demographic information (gender and home language) and pain-related information. Levene's test for variance and *t*-test for the mean between participants' total score were used for two independent groups. A *p*-value < 0.05 was considered statistically significant. Cohen's d was used as a test for independent sample effect sizes considering *d* = 0.2 as small effect, *d* = 0.5 as medium effect, and *d* = 0.8 as large effect [34]. When there were more than two independent groups (e.g., duration of pain, do you know a friend with pain, or have you been in a hospital for of pain) the ANOVA test was used and a *η*2 = 0.01 was considered as a small effect, *η*2 = 0.06 as a medium effect, and *η*2 = 0.14 as a large effect [34]. The one-way ANOVA test was used to investigate whether the school, the participants were attending had an influence on their total mean COPI score.

## 3. Results

A total of 119 children, with a mean of 17 (SD = 2.88) children per school participated. Table 1 summarises the demographic and pain-related information of the participants. Many participants indicated that they were proficient in more than one language (indicating bi- or multilingualism). Most participants chose to complete the questionnaires in English (61%), followed by isiXhosa (30%). and Afrikaans (9%). Notably, 8% of the participants reported persistent pain, and many were aware of others (friends or family) in pain.

### 3.1. Primary Objective: COPI Scores

Table 2 provides the total mean score, with SD for the adapted COPI, as well as the distribution and mean SD for each of the COPI items. Items 3, 4, and 5 had the highest mean scores, while items 8 and 9 had the lowest mean scores.

### 3.2. Secondary Objectives: Demographic and Pain-Related Information

There was no statistical difference in the total mean score of the COPI for any of the demographic and pain-related information variables (Table 3). The difference between the two means of each demographic variable (Cohen's *d* test for independent samples effect sizes) ranged between very small (*d* = 0.2) and medium (*d* = 0.5) effects. There was no significant difference in the COPI total mean score between the three groups experiencing pain for 1 week, 1 month, or > 3 months (*p*=0.533). The duration of how long pain lasted in the past had a small to medium effects (*η*^2^=0.012) on participant respective to total mean scores.

**Figure 1:**
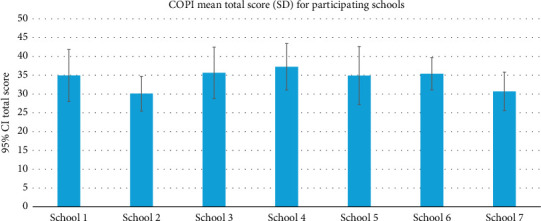
The COPI mean total score of participants for each of the 7 participating schools.

The children of seven schools participated. The total mean score of the COPI based on the responses of participants per school is presented in Figure 1. Participants from School 4 scored the highest on the COPI and participants from Schools 2 and 7 scored the lowest. The one-way ANOVA suggests a significant difference between the total scores of the seven schools (*p*=0.005). A post-hoc Bonferroni procedure suggests that there is a significant difference in the total mean scores of participants who have attended Schools 2 and 4 (*p*=0.046) and Schools 4 and 7 (*p*=0.025). Furthermore, the ANOVA's eta-squared *η*^2^=0.153 suggests that the school participants attended had a large effect on their respective total scores.

## 4. Discussion

The study aimed to determine 12-year-old primary school children's concept of pain using a cross culturally adapted COPI. The participants achieved an average score of 61% on the COPI. The score indicates that their concept of pain was partially aligned with contemporary pain science, meaning that the participants had an emerging understanding of the factors that influence the pain experience. The participants were uncertain about some COPI items, which indicates that they may have some knowledge gaps, such as the relationship between pain and tissue damage, and the value of knowledge about pain. However, participants also demonstrated pain knowledge strengths such as the purpose of pain and role of distraction in coping with pain. Published studies on COPI has focussed on its validation and not on its use in practice [7, 31]. The current study contributes to the body of evidence by analysing children's concept of pain, based on the COPI. Our analysis considered the three components of the concept of pain, namely knowledge about the purpose of pain, the biology of pain, and factors influencing pain.

### 4.1. The Purpose of Pain

Pain is essential for survival, since it acts as the body's internal alarm system that is activated to protect the individual [43]. Most participants (63%) agreed that the purpose of pain is as a warning signal for protection (Item 5). Congruently, Australian children [30] (8–12 years old) with and without pain, expressed awareness of the purpose of pain as a protection and a warning signal in a qualitative study exploring the concept of pain in children. Congruent with contemporary pain science, the above two cohorts conceptualised pain as a protective mechanism [35], albeit the difference in study design and sample population.

### 4.2. Biology of Pain

A key message of contemporary pain science is that pain is not a definite indication of tissue, but that it is multifactorial. Our participants had some difficulty conceptualising the relationship between pain and tissue damage as indicated by their variant responses to the four proxy-COPI items such as 7, 11 and 12, and particularly Item 9 which had the lowest overall score. The participants in Salvat et al. [18] who were also a school-based population of similar age (8–17 years), indicated similar uncertainties. The belief that pain is an indication of tissue damage may be a contributor for acute pain becoming chronic pain [36, 37]. It is therefore important that children be made aware of the different factors that play a role in pain awareness, and how the nervous system processes information from the body tissues [37].

Most of our participants were unaware of the role of the brain in pain processing as evident from their responses to items 6, 10 and 14 (43.5%, 35.91%, and 42.8%, respectively). Our findings corroborate with those of an Australian cohort [7] where 49% of the participants were aware of the role of the brain in pain processing. The above study (Pate et al.) [7] also used the COPI in English, in the age range of 8 to 12 years, which could explain the similarity, but their participants were children who presented for pain care at a hospital. On the other hand, in an 8–17 years old Spanish cohort [18], only 12.65% of the participants were aware of the role of the brain in pain processing, but this result was based on their completion of the COPAQ, in Catalan, in the setting of one large school. In the current study, the participants were multilingual and were provided with the option to complete the COPI in English, Afrikaans or Xhosa. The findings from Salvat et al. [18] and Pate et al. [7] were questionnaire validation studies, whereas our study focussed on the outcomes of a cross-sectional study. The study design, data collection instrument used, language of administration and the setting of recruitment (hospital or school) may have played a role in each study's findings. Differences in culture and beliefs about pain, as well as different school curriculums and educational approaches, may also explain the variations in pain concepts in the different study settings [30]. Nonetheless, understanding the role of the brain to protect the individual may help to limit negative thinking about pain. Additionally, understanding that the brain can become overprotective is fundamental to develop appropriate coping mechanisms for pain [35, 38]. The role of the brain in pain processing, as well as the weak relationship between pain and tissue damage, is important learning considerations in pain education initiatives for children to facilitate children's understanding of how biology, thoughts, emotions, experience, beliefs, social and environmental factors can influence pain [37].

### 4.3. Factors That Influence Pain

Knowledge on factors that influence pain is explored in COPI items 1-4, 8 and 13. Participants were cognizant about the value of participating in enjoyable activities when experiencing pain. However, about half were unsure/unaware about the influence of emotional factors such as stress and sadness on pain (Item 1 and Item 2). Experts agree that it is important for children to be aware of how emotions pain and vice versa [10, 39]. Learning about pain increased children's awareness about the biopsychosocial nature of pain, thereby developing empathy towards others in pain [14]. Pate et al. [30] in a qualitative study found that children with persistent pain expressed greater awareness about the emotional component of pain when compared to children without chronic pain, and Ebrahimpour et al. [40] confirm this finding. Ebrahimpour et al. [40] established that children associate pain with external (e.g., cuts and injections), internal (e.g., medical conditions or disease) and emotional factors. Since many of the participants in our study were aware of a family or friend in pain, it would be worthwhile to explore their thoughts and feelings about others in pain. To develop a greater awareness about the relationship between pain and emotions, school-based pain education initiatives can explore the interaction between thoughts, feelings (emotional), physical sensations, and behaviour/actions. Such initiatives may mitigate the interaction between pain and school-based anxiety [3]. Learning about emotional aspects of pain may be instrumental in shaping responses to pain, building resilience towards pain, and developing empathy towards individuals who has pain [6, 39]. About 65% of participants were knowledgeable that body movement can be helpful to alleviate pain. This is encouraging since moderate exercise has been found to be protective of developing spinal pain [41]. Therefore, children can benefit from the knowledge that various factors may influence pain, such as the emotional impact of pain and the benefits of exercise for psychological and physical well-being [38].

### 4.4. Learning About Pain

Participants' knowledge regarding the influence of cognition on pain varied. Most of them understood that distraction has an influence on pain (Item 3). However, participants were unconvinced that learning about pain would influence their pain experience, since only one third agreed with the statement (Item 8). This finding corresponds to the findings of Pate [7], where only 36% of an Australian cohort with pain agreed that learning about pain can be a strategy to feel less pain. Exploration of our participants' perspectives regarding learning about pain would have been beneficial, since about 8% reported having persistent pain and 19% reported current pain. Future pain education opportunities need to explore children's willingness and motivation to participate in pain education activities. To enhance children's participation, it is important that pain education initiatives must be engaging, multimodal and age appropriate to optimise children's learning about pain. Children in Pate et al. [30] enjoyed discussing pain scenarios, which may indicate a preference for active learning as opposed to passive learning strategies. Ickmans et al. 2022 [6] reviewed the literature on pain education interventions for children and offer several educational options for child appropriate pain education interventions such as games, movies, videos, comic books, stories, metaphors and visual learning material [6, 42]. For example, PNE4Kids [37] and the learning about pain comic book [43] are child-specific pain education interventions that are freely available on the internet. PNE4Kids covers the neurophysiology of pain and how it relates to everyday experiences. The pain comic book explains pain as the body's alarm system, how the neurophysiology of pain works, as well as the cognitive, emotional and behavioural factors that influence pain. Louw et al. [11] showed that follow-up education sessions, instead of single education session, are valuable to consolidate knowledge and to scaffold learning, based on mastery of the often-complex pain information. However, the interventions mentioned above may need to be adapted to be appropriate for the intended implementation setting. The abovementioned principles can guide the development of pain education initiatives that is appropriate and feasible within the school setting in order to facilitate cognitive development about pain.

The results of our study indicate that the participants would benefit from pain education, to strengthen their knowledge base, and to address their knowledge gaps about pain. Having a concept of pain that is aligned with contemporary pain science may positively impact children in future as adolescents and adults [6]. Such positive influences include promotion of recovery, resilience and adaptive pain behaviours [10]. For example, Louw et al. [11] found that in the long term children with chronic pain missed fewer days in school and used less medication after participating in a PNE, while the control group, who participated in biomedical pain education, did not report similar benefits. Therefore, improved pain literacy can have positive effects in health-care utilisation and improve coping strategies for pain. Children spend a significant amount of time at school as a social and learning environment, making schools an optimal place to reach many children, to empower children with pain-related knowledge [42, 44].

### 4.5. Socioenvironmental Factors

Demographic factors and pain-related information did not influence participants' concept of pain in the current study. This finding contrasts with existing evidence that personal pain experience and experience with family members with pain influence the concept of pain [6, 19, 30]. However, the results about correlation in our study should be interpreted cautiously, since the investigation about the possible relationships between these factors and the concept of pain was a secondary objective, and the study was not sufficiently powered to investigate these relationships. Additionally, the participants enrolled on a voluntary basis, which could introduce self-selection bias, and, therefore, may have created sample homogeneity [26]. The measurement instruments may have not been sufficiently sensitive or responsive to indicate differences, since the psychometric properties of the COPI in the SA context have not yet been established [33].

A potentially meaningful finding is the differences in the participants' concept of pain based on the school they attended. The school participants attended may be representative of socioenvironmental factors related to the community the child lives in. Our study did not explore the differences in social, cultural, environmental or educational factors that may have influenced the participants' concept of pain; therefore, there remains an impetus to broaden our understanding the influence of socioenvironmental context on the concept of coping with pain [40], in the African context. Social, cultural, educational and environmental contexts are foundational in a child's understanding and expression of pain [10]. Indeed, children in Pate et al. [30] acknowledged that they learnt about pain from school, family, social media and television. Other factors that influence a child's concept of pain include parental cognitive, affective and behavioural responses to pain [6], the child's stage of growth and development, psychological age, cognitive development and education level [45].

Social aspects influencing pain have not received as much attention in research when compared to the biological and psychological aspects. However, Kapos et al. [9] reviewed the literature about the impact of social factors on pain concept, expression and management. They developed a multilevel framework to synthesise the social aspects of pain into an intrapersonal level (e.g., roles, relatedness and support), a community level (e.g., social categories and group belonging) and a societal level (e.g., political, economic and cultural systems) [9]. There is a complex interrelationship between these levels throughout the life course [9, 19]. For example, while culture is passed on primarily by the family to the developing child, later in life various social institutions influences culture (e.g., school, church and/or other religious or community institutions), which have a major impact on the child's psychological, emotional and cognitive development [6]. Congruently, two systematic reviews found moderate to preliminary evidence for differences in the pain processing, the concept of pain, pain coping strategies, illness perception and pain attitudes in different populations [19, 27].

SA has diversity of cultures, ethnicities, religions and 11 official languages. Nortjé and Albertyn [20], in a qualitative study, found that culture influences the meaning of pain in different cultural groups in SA. In the Nguni culture (inclusive of isiXhosa), pain is linked to a physical source and an imbalance in the body and is viewed as a form of communication from ancestors. There are also indications of gender differences in how pain is expressed in the Nguni and Sotho cultures and children in these cultures are taught to show resilience in enduring pain [20]. Language and culture are often seen as interrelated [46], and language is often used as a proxy for culture or ethnicity. However, this proxy becomes blurred in a multilingual society such as SA, as can be seen in that many of our participants were bilingual or multilingual. The influence of multilingualism in response to COPI items needs further investigation. Our findings indicate the importance of considering culture when planning pain education initiatives for children.

### 4.6. Strengths and Limitations

To the knowledge of the authors, this study is the first to explore children's concept of pain in Africa and the first to elaborate on children's concept of pain using the COPI. The sampling of schools in different geographical regions provided diversity in language use and environmental factors. The study identified knowledge gaps and strengths in participants' concept of pain, to inform an appropriate school-based pain education programme for the region. As part of the study, the COPI was adapted for the SA setting and was translated in two additional languages (Afrikaans and isiXhosa), to be available for further use in SA.

While the findings provided useful insights into the concept of pain in children in this geographical area, care should be taken when applying the results to other geographical locations or age groups. The study relied on voluntary participation and self-report, which can lead to response biases and limitations inherent in self-report methods [26]. For example, in response bias, the participants may not be representative of the overall population, and in response bias, responders may differ systematically from nonresponders [21]. Therefore, the findings cannot be generalised to SA children but should be seen as preliminary and exploratory findings. The study sample size was small, and the study did not analyse the sources of pain information, or the influence of cultural, social and environmental factors that may inform participants' concept of pain. Due to COVID-19 restrictions, and resultant infection control measures, we could only distribute a limited number of questionnaires per school. We did not implement an electronic survey due to the risk that it may systematically exclude potential participants who did not have access to electronic devices. As a result, more research with powered and varied sampling is required. When sufficiently powered studies are conducted, we recommend that additional sensitivity analysis should be conducted, to verify the findings. We further acknowledge that, also due to COVID-19 restrictions, a teacher had to assist in the recruitment of the participants and explain the inclusion criteria to the children. The exclusion criterion of children who had received therapy from the PI was not verified. The above aspect may have influenced participants' selection. The psychometric properties of COPI in SA should be evaluated, and translation into the other SA languages is needed. Additionally, we recommend a qualitative study to understand participants' responses to COPI items and to gain deeper insights into children's conceptualisation of pain. We recommend mixed-methods research to determine and to explore the influence of social factors on the development of the concept of pain in different contexts.

## 5. Conclusion

The participants' concept of pain as measured with the cross culturally adapted COPI indicated that they had emerging knowledge on the purpose of pain, the biology of pain and factors that influence pain. The participants had knowledge gaps, and strengths can be used to develop and implement a tailored pain education intervention for them. Such an intervention would need to be engaging, multimodal and age appropriate. While demographic factors and pain-related information did not influence participants' concept of pain in this study, there are indications that socioenvironmental factors may have influenced their concept of pain. Further research to explore these contextual factors is suggested.

## Figures and Tables

**Table 1:** Participants' demographic and pain-related information.

Demographic variable	Participant		Pain-related information	Participant	
	*n*	%		*n*	%
isiXhosa proficient	60	50.4	Currently experiencing pain	22	18.5
English proficient	62	52.1	Has experienced pain before	110	92.4
Afrikaans proficient	34	28.6	Duration of pain: 1 week	68	57.1
Other languages proficiency (Russian *n* = 1 and Chinese *n* = 1)	2	1.7	Duration of pain: 1 month	31	26.1
Boys	49	41.2	Duration of pain: > 3 months	10	8.4
Girls	70	58.8	Consulted a doctor for pain	75	63
			Been in hospital for pain	50	42
			Know a friend who has pain	56	47
			Have a family member who has pain	75	63

**Table 2:** Summary of participants' responses to the adapted COPI items.

Answer options		Strongly disagree		Disagree		Unsure		Agree		Strongly agree		M	SD
Likert scale score (0–4)		0		1		2		3		4			
Nr	Item	*n*	%	*n*	%	*n*	%	*n*	%	*n*	%		
1	Feeling stressed, worried and upset can make you feel more pain.	8	6.8	16	13.4	35	29.4	40	33.6	20	16.8	2.4	1.122
2	Feeling sad or down can make you feel more pain.	6	5	17	14.3	39	32.8	43	36.1	14	11.8	2.35	1.030
3	Being distracted and keeping busy can make you feel less pain.	3	2.5	14	11.8	18	15.1	55	46.2	29	24.4	2.78	1.026
4	Doing something you enjoy can make you feel less pain.	3	2.5	8	6.7	12	10.1	46	38.7	50	42	3.1	1.007
5	Pain is a warning message that the body needs to be protected.	2	1.7	9	7.6	33	27.7	44	37	31	26	2.78	0.976
6	Feeling pain for a long time can make the brain more sensitive to warning messages.	1	0.8	15	12.6	51	42.9	28	23.5	24	20.2	2.5	0.982
7	You can feel a lot of pain even when an injury is small.	7	5.9	18	15.1	15	12.6	56	47.1	23	19.3	2.59	1.138
8	Learning and understanding about pain can help you to feel less pain.	11	9.2	18	15.2	53	44.5	29	24.4	8	6.7	2.04	1.020
9	You can have an injury and feel no pain.	22	19	32	26.9	20	16.8	30	25.2	15	12.6	1.87	1.327
10	Activities in the brain can make pain better or worse.	3	2.5	11	9.5	62	52.1	32	26.7	11	9.2	2.31	0.861
11	You can feel a little bit of pain even when an injury is big.	14	12	21	17.6	21	17.6	50	42	13	10.9	2.23	1.210
12	You can feel pain even after an injury has healed.	8	6.8	21	17.6	26	21.8	45	37.8	19	16	2.39	1.151
13	Pain usually feels better if you move your body a little bit more each day.	12	3.4	12	10.1	25	21	55	46.2	23	19.3	2.68	1.008
14	The brain works through and processes a lot of information before you feel pain.	9	3.4	9	7.6	55	46.2	42	35.2	9	7.6	2.36	0.861
**Total score out of 56**												**34.4**	**6.49**

*Note:* The bold text indicates the summary scores for the M and SD for the total number of participants.

Abbreviations: M = mean, SD = standard deviation.

**Table 3:** Relationship between the COPI total scores and demographic and pain-related variables.

Variable	COPI total mean score		*t*-test (*p* < 0.05)	Cohen's D
	Boy	Girl	*p*	*d*
Gender	34.184	34.529	0.777	0.053

	**No**	**Yes**		

Self-complete	34.220	36.200	0.358	0.305
isiXhosa	35.153	33.633	0.203	0.235
English	34.018	34.726	0.554	0.109
Afrikaans	34.529	34.029	0.706	0.077
Went to hospital for pain	34.913	33.660	0.301	0.193
Saw a doctor for pain	35.455	33.760	0.170	0.262
Experienced pain before	33.778	34.436	0.771	0.101
Have family member with pain	34.636	34.240	0.749	0.061
Know a friend with pain	34.476	34.240	0.874	0.029

## Data Availability

The data that support the findings of this study are available from the corresponding author upon reasonable request.

## Supporting Information

STROBE statement—Checklist of items that should be included in reports of cross-sectional studies.
